# Frameless Co-Registration of Biplane 2D Digital Subtraction Angiography Whole Frames and 3D Rotational Angiography-Based Cone-Beam Computed Tomography Angiogram on Dedicated Software for Stereotactic Radiosurgery of Cranial Vascular Malformations

**DOI:** 10.7759/cureus.27983

**Published:** 2022-08-14

**Authors:** Kazuhiro Ohtakara, Takashi Izumi, Kuniaki Tanahashi, Takeshi Kamomae, Kojiro Suzuki

**Affiliations:** 1 Department of Radiation Oncology, Kainan Hospital Aichi Prefectural Welfare Federation of Agricultural Cooperatives, Yatomi, JPN; 2 Department of Radiology, Aichi Medical University, Nagakute, JPN; 3 Department of Radiology, Nagoya University Graduate School of Medicine, Nagoya, JPN; 4 Department of Neurosurgery, Nagoya University Graduate School of Medicine, Nagoya, JPN; 5 Department of Neurosurgery, Gifu Prefectural Tajimi Hospital, Tajimi, JPN

**Keywords:** less invasive, frameless, image co-registration, 3d rotational angiography, stereotactic radiosurgery, dural arteriovenous fistula, digital subtraction angiogram, cone-beam computed tomography angiogram, arteriovenous malformation

## Abstract

Purpose: Given its high spatial resolution and vasculature selectivity, the cone-beam computed tomography (CT) angiography (CBCTA) image acquired by selective 3D rotational angiography (3DRA) is the most suitable 3D image for the target definition of stereotactic radiosurgery (SRS) for intracranial arteriovenous malformations (AVMs) and dural arteriovenous fistulas (DAVFs). Furthermore, the relatively low temporal resolution of 3DRA-based CBCTA can be complemented by the stereotactic co-registration of orthogonally paired 2D dynamic digital subtraction angiography (2D-DSA). The integration of 2D-DSA, which is usually limited to one or a few frames for each projection, into CBCTA and/or planning CT can be achieved only by catheter-directed angiography on the day of SRS via a dedicated image localizer under rigid frame fixation to the skull, which imposes substantial burdens on patients. This study aimed to demonstrate a novel, convenient, and significantly less invasive method for the frameless co-registration of biplane 2D-DSA whole frames and CBCTA on commercially available dedicated software, namely, Brainlab^®^ Elements (Brainlab AG, Munich, Germany), and present its prerequisite for successful image fusion.

Technical Report: Elements have afforded the following functionality: A 3D vasculature image is automatically extracted as a floating image from any 3D image series containing vascular details and then subsequently co-registered manually and automatically to a selected frame pair of 2D-DSA with a six-degree-of-freedom rigid registration. As a preclinical feasibility study, two anonymous image datasets from patients harboring cerebral AVM and transverse-sigmoid (TS) DAVF were used to verify the accuracy and practicality of Elements for the frameless co-registration of 2D/3D vascular images, particularly on the assumption of clinical workflow for the target delineation of SRS planning. The use of ordinary unsubtracted CBCTA resulted in the insufficient extraction of abutting vessels or vessels that are in close proximity to bony structures, particularly in the case of TS-DAVF, where the fistulous pouch and the affected venous sinuses were adjacent to the cranial bone. By contrast, the amount and selectivity of vasculatures and the accuracy of subsequent image fusion were significantly improved from the subtracted CBCTA. The integration of CBCTA into dynamic 2D-DSA allowed the simultaneous review of both image information by sharing any concerning point and 2D or 3D structures under a common 3D coordinate.

Conclusions: Elements enable the clinically useful frameless co-registration of biplane 2D-DSA whole frames into CBCTA, for which the routine acquisition of both subtracted and unsubtracted CBCTA axial images for ordinary diagnostic purposes is an indispensable prerequisite for successful image fusion and further widespread application. This frameless integration of the 2D/3D angiogram would dramatically enhance both the frame-based and frameless SRS workflow and circumstances by allowing users to forward SRS planning well in advance before SRS, along with the omission of invasive angiography on the day of SRS, and would broaden the implementation of frameless SRS. Furthermore, the comprehensive alternating interactive review of the 2D/3D integrated angiogram leads to a more in-depth quasi-4D understanding of the affected angioarchitectures compared with the separate viewing of each image.

## Introduction

Stereotactic radiosurgery (SRS) is an indispensable, definitive treatment option for patients with symptomatic arteriovenous malformation (AVM) and/or dural arteriovenous fistula (DAVF), which are not amenable to direct surgery or endovascular embolization alone [1,2]. Traditionally, the stereotactic co-registration of one or a few representative frames of orthogonally paired 2D digital subtraction angiography (2D-DSA) into a 3D setup, as well as planning images via a dedicated image localizer under rigid frame fixation to the skull, has been a standard prerequisite procedure for target delineation in SRS for intracranial AVM and DVAF. However, the use of frame fixation for a prolonged period and performing follow-up angiography on the day of SRS impose substantial burdens on patients [1].

Among the 3D imaging techniques for visualizing affected angioarchitectures, cone-beam computed tomography (CT) angiography (CBCTA) acquired by C-arm-based selective 3D-rotational angiography (3DRA) is recognized as the most suitable foundation for target definition in terms of spatial resolution, can visualize tiny lesions and/or fine caliber vessels, and distinguish nidus segmentation from different feeding arteries [1,3-17]. With the advent of image-guidance systems and the application of CBCTA, a frameless SRS workflow has been adopted in an increasing number of institutions implementing CyberKnife, conventional linac, and Gamma Knife, where treatment planning is usually completed by the day before SRS, thus allowing for the omission of angiography on the day of SRS [3-5,11,13-16,18]. However, in this frameless workflow, 2D-DSA cannot be directly integrated into a 3D planning image such as CBCTA and/or non-contrast planning CT and is only available as a reference image. Although target outlining based on CBCTA alone may be adequate for cases with a relatively simple lesion configuration and relevant angioarchitecture [3,5,7,8,11], the relatively low temporal resolution of CBCTA likely becomes problematic in cases with a complex vasculature, such as an irregularly shaped and/or diffuse nidus and rare localization. Biplane 2D-DSA, including oblique angulation with the adequate temporal resolution, provides dynamic, comprehensive, and panoramic views of the affected angioarchitecture, whereas paired 2D-DSA alone is inadequate for 3D target outlining owing to its low spatial resolution [5-7].

To redeem the low temporal resolution of CBCTA and to expand the applicability of frameless radiosurgery, the frameless integration of 2D-DSA (whole frames, if possible) into CBCTA is required [5,10]. Thus far, the frameless integration of 2D-DSA into CT and/or 3D vascular images has been attempted using various approaches, such as the implantation of fiducial markers or originally developed software prototypes [13,14]. Recently, an upgraded version of dedicated planning-subsidiary software Brainlab® Elements (Brainlab AG, Munich, Germany) has provided the frameless co-registration functionality of 3D vascular images and orthogonally paired 2D-DSA [19]. To the best of our knowledge, Brainlab® Elements has so far been the only commercially available software enabling frameless 6 degree-of-freedom (6DoF) co-registration of 2D-DSA whole frames into any stereotactic 3D vasculature-visible image. Thus, we examined the accuracy and practicality of this software by using actual clinical images in a preclinical feasibility study to verify whether the frameless co-registration of 2D-DSA and CBCTA is feasible in a clinical setting.

The synopsis of this study was presented at the 13^th^ annual meeting of the Japan Radiosurgery Society held online on February 5, 2022.

## Technical report

Clinical cases

**Figure 1:**
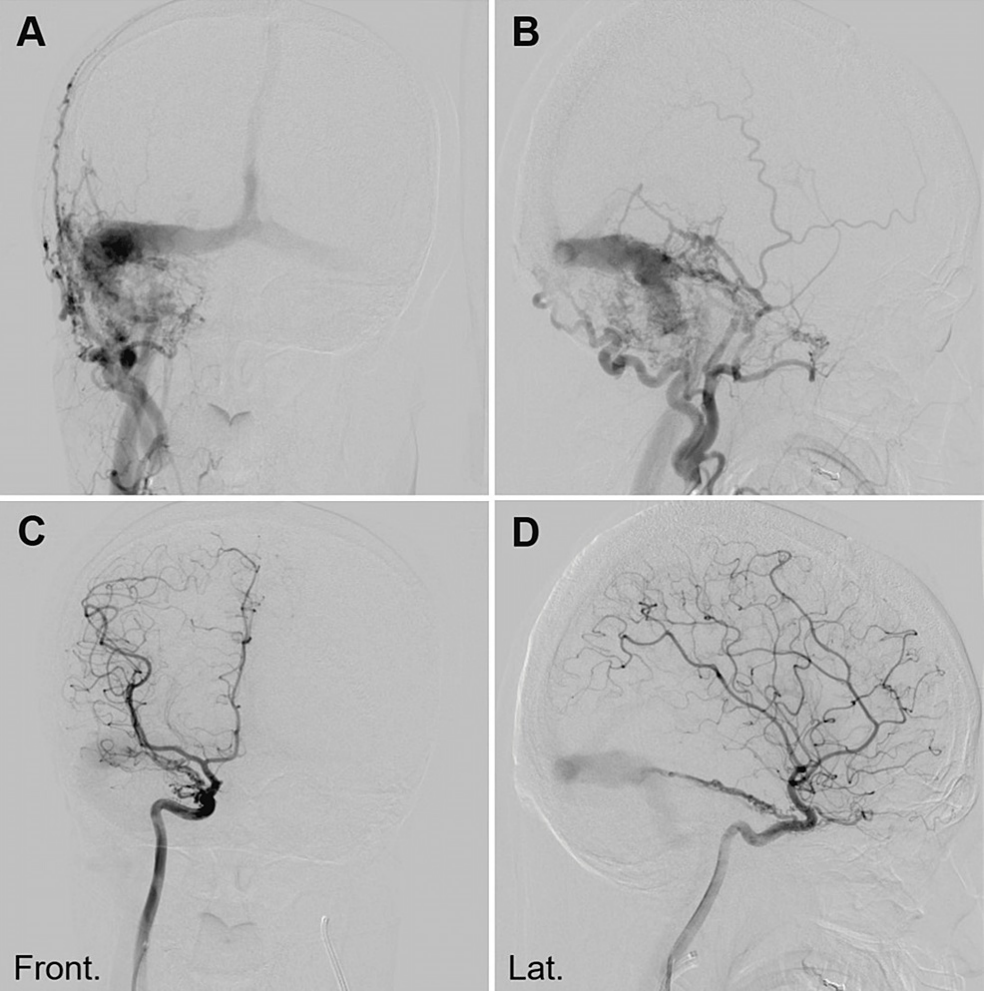
Digital Subtraction Angiogram (DSA) Showing Right-Sided Transverse-Sigmoid Dural Arteriovenous Fistula (TS-DAVF). A, B: right external carotid artery (ECA) injection; C, D: right internal carotid artery (ICA) injection; A, C: frontal view; B, D: lateral view; Front: frontal view; Lat.: lateral view.

**Figure 2:**
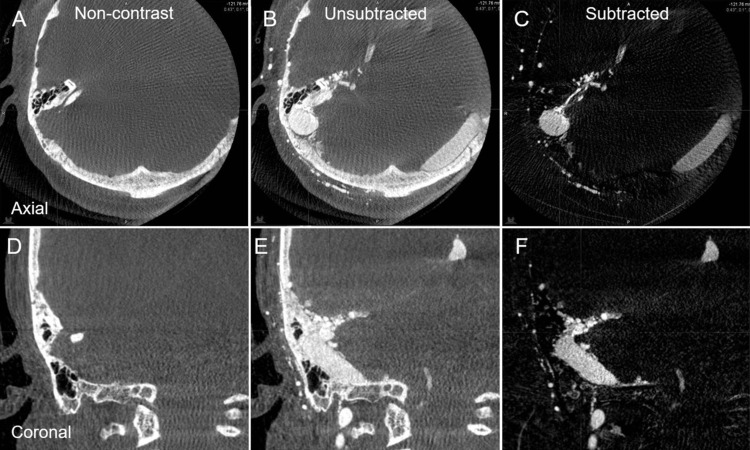
Cone-Beam Computed Tomography Angiogram (CBCTA) of Right ECA Injection in the Case with Right TS-DAVF. A–C: axial views; D–F: coronal views; A, D: pre-contrast images; B, E: post-contrast unsubtracted images; C, F: post-contrast subtracted images. The reconstructed images of both unsubtracted and subtracted CBCTA along with pre-contrast CBCT clearly show the 3D angioarchitecture in detail.

The anonymous image datasets from two representative cases with AVM in the left occipital lobe and DAVF in the right TS junction, respectively, previously treated with stereotactic radiosurgery (SRS) combined with transarterial embolization, were selected in this study and imported into Elements. The used images include the whole frames (six [arterial phase] and three [venous phase] frames per second) of orthogonally paired 2D-DSA (Figure 1), the reconstructed unsubtracted and subtracted axial images of CBCTA obtained by 3DRA with internal carotid, external carotid, and/or vertebral artery injections (Figure 2), non-contrast planning CT images, and the original axial images of 3D time-of-flight (TOF) magnetic resonance angiography (MRA) without contrast.

Catheter-directed angiography was performed on the Siemens Artis Q biplane system (Siemens Healthineers, Erlangen, Germany) in which subtracted CBCTA was routinely programmed.

Principle and algorithm of Brainlab® Elements

**Figure 3:**
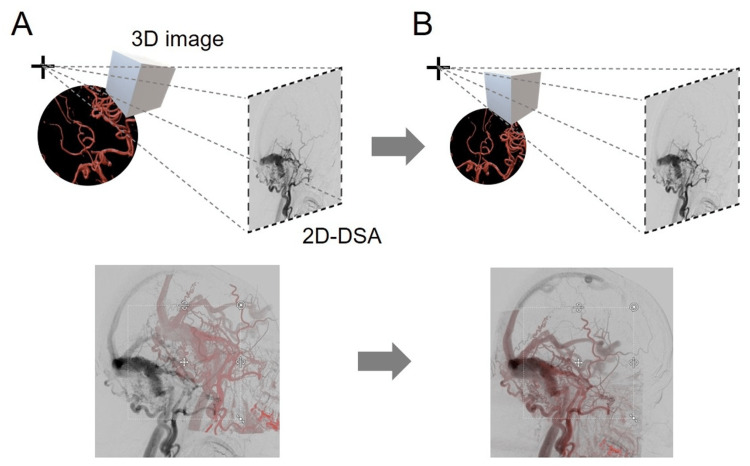
Schematic Illustrations of the Algorithm for the Co-Registration of 2D/3D Vasculature Images by Brainlab® Elements. A: pre-registration; B: post-registration. Elements automatically pick out the 3D vascular component from any 3D vasculature-visible image as a floating image and then superimposes the 3D vascular image onto a selected frame pair of biplane 2D-DSA. After the superimposition, the position, scale, and three-axis rotation of the 3D vascular image layer is manually adjusted to overlap as mutually as possible, which enhances the accuracy of subsequent automatic co-registration, and finally the six-degree-of-freedom (6DoF) vascular image fusion is accomplished automatically in a rigid registration manner.

In Elements, the 3D vasculature component can be automatically extracted as a floating image from any 3D image series containing vascular details and then subsequently superimposed on a selected frame pair of biplane 2D-DSA [19]. Thereafter, the users manually correct the misalignment of the floating 3D image relative to the underlying 2D-DSA by adjusting the position, dimension, and/or rotation of the floating image so that these parameters overlap as mutually as possible. Elements then automatically co-register the data with a six-degree-of-freedom rigid registration (Figure 3).

The resultant 2D/3D angiogram fused dataset can be integrated into other 3D images, such as planning unenhanced CT in alignment with the bony structure of unsubtracted CBCTA.

Frameless co-registration of MRA and CBCTA

**Figure 4:**
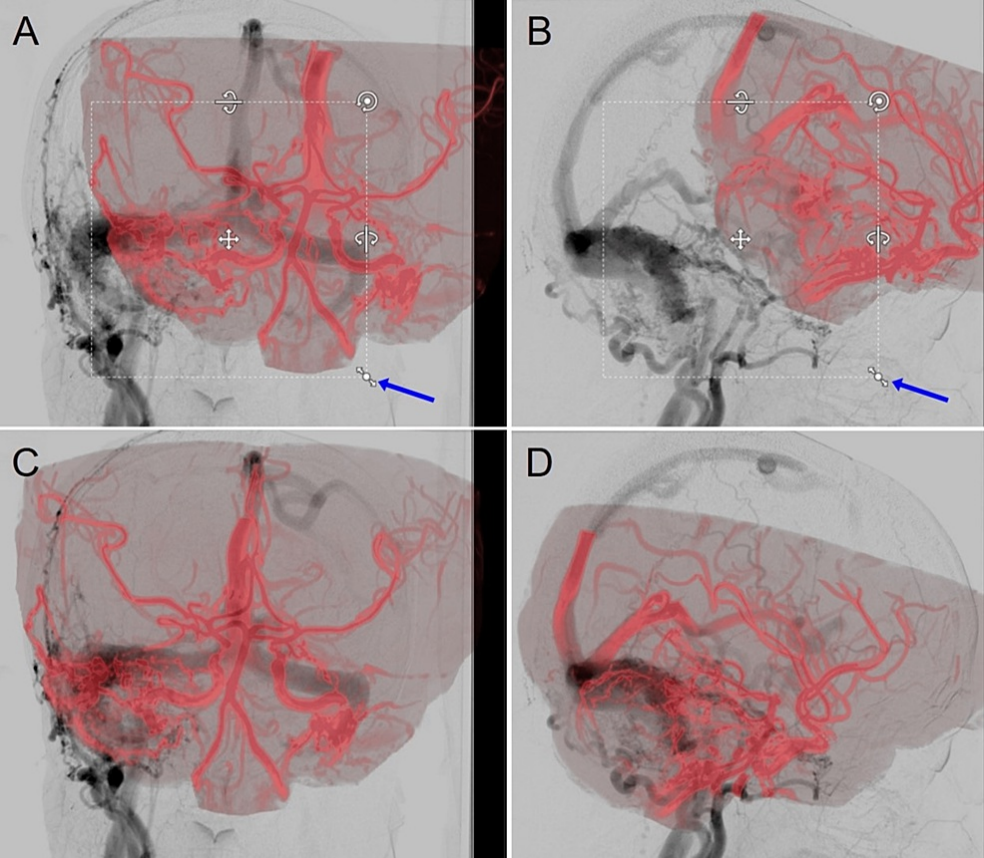
Frameless Co-Registration of Magnetic Resonance Angiogram (MRA) into 2D-DSA via Elements. A, B: Crude vasculature images (red color) automatically extracted from 3D time-of-flight (TOF) MRA original axial images on the background of paired 2D-DSA. Users can then manually adjust the position, scale, and three-axis rotation of the 3D vascular image layer (arrows) to better match the underlying DSA images and subsequently to enhance the accuracy of automatic 6DoF fusion. C, D: After final automatic fusion.

The automatic extraction of 3D vasculature images from 3D-TOF MRA and subsequent image fusion into 2D-DSA pairs was performed without any specific difficulty, but the selectivity and crispness of the vasculature configuration were admittedly inferior to those of CBCTA (Figure 4).

Frameless co-registration of orthogonally paired 2D-DSA whole frames and CBCTA

**Figure 5:**
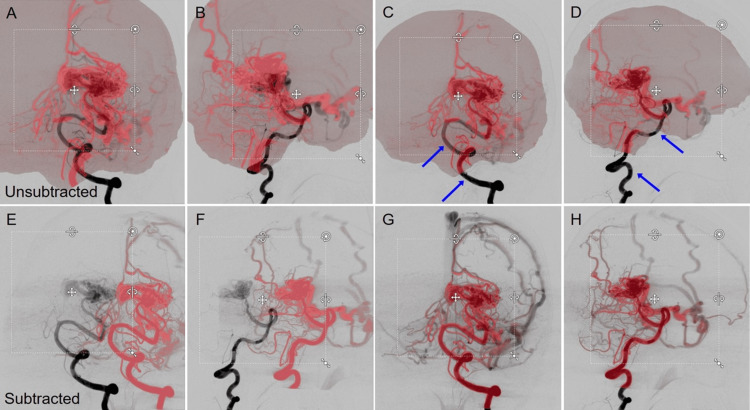
Comparison of Unsubtracted and Subtracted CBCTA Co-Registered with 2D-DSA for Left Occipital Arteriovenous Malformation. A–D: unsubtracted CBCTA; E–H: subtracted CBCTA; A, B, E, F: pre-fusion; C, D, G, H: post-fusion. Several defects on the major vessels (arrows) adjacent to the cranial bone are conspicuous on the unsubtracted CBCTA, whereas better vasculature visualization, including early filling veins, is demonstrated on the subtracted CBCTA.

In the case of parenchymal deep-seated AVM, the use of the usual unsubtracted CBCTA resulted in the insufficient extraction of abutting vessels or vessels that are in close proximity to bony structures, particularly the cranial base, where several defects of the major arteries were observed. However, the 2D/3D co-registration was successfully completed. Alternatively, the use of subtracted CBCTA significantly improved the amount and selectivity of the extracted vasculature images (Figure 5).

**Figure 6:**
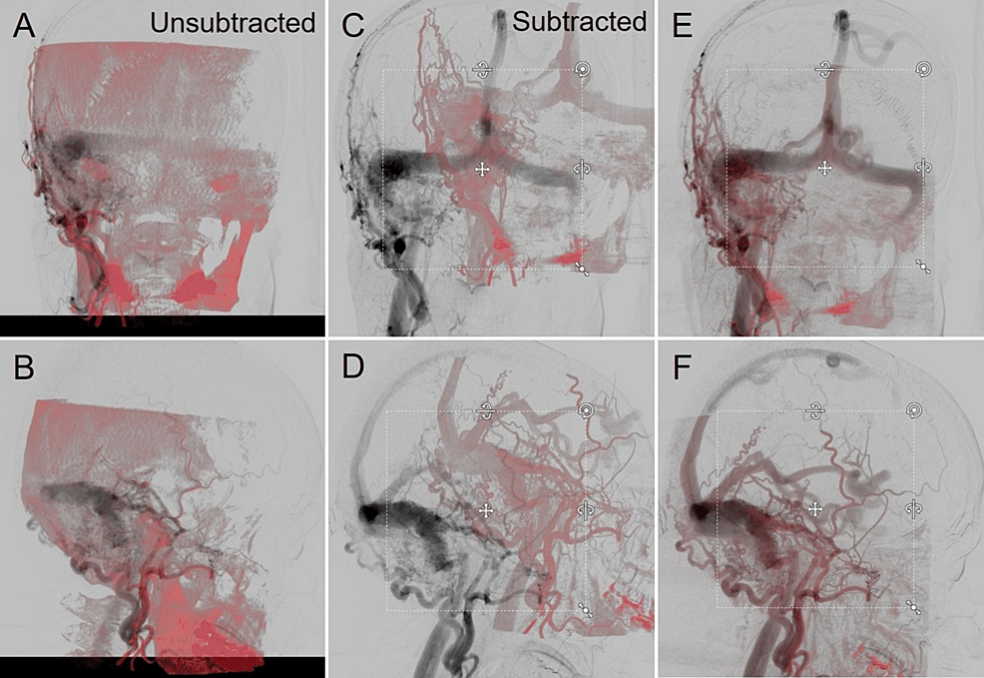
Comparison of Unsubtracted and Subtracted CBCTA on the Results of Co-Registration with 2D-DSA for Right TS-DVAF. A, B: unsubtracted CBCTA; C–F: subtracted CBCTA (C, D: pre-fusion; E, F: post-fusion). Note the difference in the selectivity of the vasculature images from each CBCTA.

In the case of TS-DAVF, the use of unsubtracted CBCTA resulted in a significantly insufficient extraction of the vasculature, where the early filling venous sinuses, including the fistulous pouch adjacent to the cranial bone, were almost indistinguishable from the background. Accordingly, automatic 2D/3D image fusion culminated in failure. On the contrary, the use of subtracted CBCTA dramatically improved the amount and selectivity of the extracted 3D vasculature from CBCTA, including the branches of the external carotid artery and the affected venous sinuses. Furthermore, the subsequent co-registration into the 2D-DSA pair was successfully executed (Figure 6).

**Figure 7:**
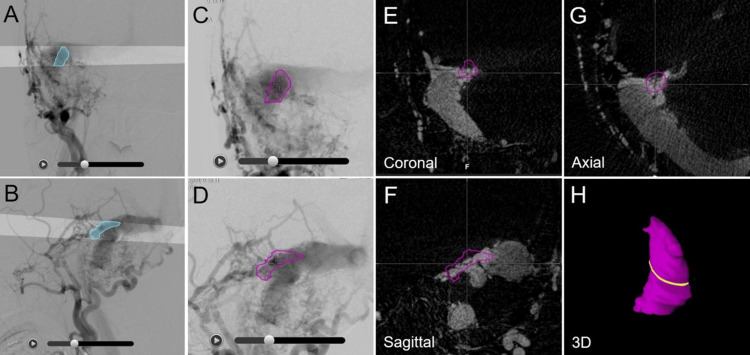
Target contouring process after 2D/3D angiogram fusion for TS-DVAF. A–D: 2D-DSA (A, C: frontal view; B, D: lateral view); E–G: CBCTA subtraction images; H: 3D view of an automatically generated 3D object (purple) based on the tentative outlining of the target on the DSA pair. A, B: Tentative 2D outlining of the dominant arteriovenous shunt portion (blue) on the 2D-DSA pair. Each highlighted region of interest projects a ray onto the other view and intersects appropriately. The user can review the entire frame of the 2D-DSA pair by scrolling through the images using sliders. This 3D structure (purple) leaves room for further modification to generate an appropriate target structure without over- or under-coverage.

The successful integration of CBCTA into dynamic 2D-DSA whole frames via subtracted CBCTA allowed the repeated and simultaneous review of both the whole frames of the integrated biplane 2D-DSA in either the forward or backward direction and the CBCTA alternately and interactively with any shared concerning point, 2D, or 3D structures under a common 3D coordinate (Figure 7).

A tentative 2D outline of the region of interest on a 2D-DSA image was automatically projected as a ray onto the other pair. If the 2D contouring pair intersecting appropriately on the 2D-DSA pair was defined once, a tentative 3D structure superimposed onto both the 2D-DSA pair and CBCTA was automatically generated. However, a crude 3D structure based on biplane 2D-DSA usually leaves substantial room for further modification of either over- or undercoverage to define the appropriate target.

Elements also enabled the 2D/3D angiogram fusion dataset to be co-registered with unenhanced CT for SRS dose planning in alignment with the bony structure of the unsubtracted CT. The unenhanced CT could also be fused with several magnetic resonance imaging (MRI) sequences that visualize the affected brain parenchyma and/or the critical structures juxtaposed to the nidus (data not shown).

## Discussion

This preclinical study demonstrated the functionality of Brainlab® Elements for the convenient and clinically useful co-registration of biplane 2D-DSA whole frames and CBCTA. Subtracted and unsubtracted CBCTA is important for achieving accurate image fusion. Whether subtracted CBCTA acquisition is routinely performed depends on the angiography equipment and device. Therefore, when performing catheter-directed angiography for routine diagnostic purposes in cases with AVM or DAVF, the acquisition of subtracted and unsubtracted CBCTA is an imperative prerequisite for further clinical application, in addition to 2D-DSA with adequate frames per second. The resulting 2D and 3D angiogram datasets can be co-registered to both the planning CT and MRI in alignment with the bony structure and brain parenchyma, respectively [3,4]. Elements also contain an image distortion correction functionality for MRI scans. Taken together, the co-registered image dataset, including unenhanced CT for dose calculation, MRI for the recognition of accurate target localization in the affected normal tissues, and 2D/3D fused angiogram for target delineation, would be adequate for SRS planning [7]. In contrast to frame-based co-registration via an image localizer, Elements enables the integration of biplane 2D-DSA whole frames, that is, time-series imagery, which allows the repeated viewing of dynamic 2D-DSA in either the forward or backward direction with 3D-DSA (CBCTA) alternately and interactively, thus leading to more in-depth quasi-4D interpretations of the affected angioarchitecture than with the separate viewing of each image or the viewing of images available in frame-based settings. Furthermore, this comprehensive review of the 2D/3D integrated angiogram would also be widely useful for all medical trainees and professionals engaged in the diagnosis and management of AVM and DAVF beyond specialists involved in SRS and might hold the notable potential to enhance the diagnostic armamentarium for various vascular disorders beyond the intracranial region.

The Elements-directed co-registration of 2D-DSA and CBCTA dramatically enhanced the treatment workflow and circumstances of both frame-based and frameless SRS [15,17]. Particularly, AVM and DAVF with complex angioarchitecture are among the most difficult cases for target definition in SRS, for which sufficient time needs to be devoted. In this regard, this 2D/3D angiogram fusion can subserve treatment planning, including target definition, well in advance before SRS and omit the need for invasive angiography on the day of SRS, thus significantly reducing treatment time and burdens on both patients and medical professionals [1]. Elements eliminated one of the remaining advantages of frame-based SRS, that is, the integrated diagnosis of both CBCTA and limited frames of 2D-DSA, and would become a tailwind for the further implementation of frameless SRS [15,19]. One of the remaining issues concerning frameless SRS has been an intra-fractional patient movement within a thermoplastic mask, for which more frequent detection and correction of head motion during treatment can be performed owing to the recent advent of bony and surface image-guidance systems (e.g., ExacTrac Dynamic® [Brainlab AG], which utilizes continuous monitoring by a thermal camera and/or frequent and timely kV image acquisition during irradiation) [20].

This study includes several limitations and needs further investigation to verify the clinical utility of frameless co-registration of 2D-DSA and CBCTA achieved by Brainlab Elements. In some cases, 2D-DSA images obtained by oblique projection with an appropriate angular degree enhance the visibility of the lesion configuration compared to the frontal and lateral projection alone. Although Elements is also applicable to any 2D-DSA pair with oblique projection according to the vendor, oblique angulation 2D-DSA pairs were, unfortunately, unavailable in this study. Moreover, this was a preclinical report with only two representative cases, and the results must therefore be validated through a large number of cases with various lesional configurations, locations, and relevant angioarchitecture, including extracranial vessels.

## Conclusions

The frameless stereotactic co-registration of orthogonally paired 2D-DSA whole frames into CBCTA can be achieved by using Brainlab® Elements, for which routine acquisition of both subtracted and unsubtracted CBCTA for ordinary diagnostic purposes in cases with AVM and DAVF is a sine qua non for successful image fusion and further broad applications. This convenient frameless integration of 2D/3D angiograms enables SRS planning well in advance before SRS and the omission of catheter-directed angiography on the day of SRS, thus dramatically enhancing the treatment workflow and circumstances and consolidating and ensuring the effective implementation of frameless SRS and frame-based SRS for AVM and DAVF.
